# Proliferation and Differentiation in the Adult Subventricular Zone Are Not Affected by CSF1R Inhibition

**DOI:** 10.3389/fncel.2019.00097

**Published:** 2019-04-02

**Authors:** Jackson Kyle, Michelle Wu, Stefania Gourzi, Stella E. Tsirka

**Affiliations:** Molecular and Cellular Pharmacology Graduate Program, Department of Pharmacological Sciences, Stony Brook University, Stony Brook, NY, United States

**Keywords:** CSF1R, microglia, PLX5622, adult neurogenesis, neural stem cells

## Abstract

Microglia are reported to have significant roles in regulating normal mammalian adult neurogenesis. There are two neurogenic niches in the adult mammal brain: the subgranular zone (SGZ) in the hippocampus, and the subventricular zone (SVZ), which makes up the lining of the lateral ventricles. While the microglia interactions on adult neurogenesis in the hippocampus have been characterized, the SVZ niche is not as well investigated. The SVZ niche is unique in that the newborn neurons migrate a much longer distance through multiple brain structures compared to newborn neurons in the hippocampus, making it more difficult to fully characterize how microglia influence this process. To examine the SVZ niche and migration pathway, we used the colony stimulating factor 1 receptor (CSF1R) antagonist PLX5622, which promotes brain wide microglia ablation. Microglia ablation resulted in no changes in the numbers of neural stem cells (NSCs), transient amplifying cells, and neuroblasts. Microglia ablation in the olfactory bulb (OB) was decreased compared to the SVZ. CSF1R inhibition had no effect on the ability of microglia to proliferate. Thus, our data suggest that microglia are not required for normal functioning SVZ adult neurogenesis.

## Introduction

In the adult mammalian brain, new neurons are continuously born from neural stem cells (NSCs; reviewed in Bond et al., 2015). These NSCs reside in two niches in the adult brain—the subventricular zone (SVZ), which forms the lining of the lateral ventricles, and the subgranular zone (SGZ) in the hippocampus. In the SVZ, NSCs give rise to transit amplifying cells, which can produce neural progenitors called neuroblasts. Neuroblasts migrate out of the SVZ niche to the olfactory bulb (OB) *via* the rostral migratory stream (RMS) following “chain migration” (Lois et al., 1996). In the OB neuroblasts mature into neurons, but few become integrated into the existing neuronal circuits. SVZ-OB neurogenesis functions in odor recognition in mammals, while hippocampal neurogenesis is important for memory and learning (Reshef et al., 2014). In pathological conditions and injury, such as ischemic stroke, stimulated NSCs proliferate in both the SVZ and SGZ (Kokaia and Lindvall, 2003; Parent, 2003). Newborn neurons from the SVZ have been reported to divert from their migration along the RMS and instead migrate towards the lesion site. However, most of these neurons do not survive long term (Arvidsson et al., 2002).

Other cell types populating the neurogenic niche microenvironment have been shown to influence adult neurogenesis, including microglia. Microglia are the resident immune cells of the central nervous system (CNS; reviewed in Li and Barres, 2018). They become activated in response to injury or infection, becoming either classically activated pro-inflammatory (M1-like), or alternatively activated anti-inflammatory (M2-like). Activated microglia release pro- and anti-inflammatory cytokines, respectively, and both pathways result in increased phagocytosis of cellular debris and pathogens (Butovsky et al., 2006). Recently many studies focus on investigating microglia before they become activated, while they remain in a “resting” state. Resting microglia continuously sample and monitor their local environment with their ramified processes that are constantly motile (Nimmerjahn et al., 2005). In this state, microglia are involved in developmental and adult neuronal pruning and synapse sculpting *via* the microglial CX3C receptor-1 (CX3CR1) interacting with the neuronal CX3C ligand-1 (CX3CL1; Paolicelli et al., 2011; Reshef et al., 2017).

Microglia have been shown to be important for adult neurogenesis. In culture experiments, microglial conditioned media promoted migration of neuroblasts (Aarum et al., 2003). Pro-inflammatory environments formed by microglia hindered and reduced neurogenesis (Ekdahl et al., 2003). However, microglia can also promote neurogenesis following alternative anti-inflammatory activation accompanied by the secretion of IL-4 (Butovsky et al., 2006; Shigemoto-Mogami et al., 2014). In the hippocampus, phagocytosis of apoptotic neuroblasts and progenitors by microglia was shown to be important for the proper functioning of local adult neurogenesis (Sierra et al., 2010). In the SVZ, however, it is not as clear. Microglia in the adult rodent SVZ do not express TREM2 (triggering receptor expressed on myeloid cells 2), a receptor involved in phagocytosis (Takahashi et al., 2005), suggesting that regular clearing of cells by microglia does not happen in the SVZ (Sierra et al., 2010; Ribeiro Xavier et al., 2015). However, injections of saporin conjugated to CD11b, a toxin which depletes microglia locally at the SVZ, resulted in decreased numbers of neuroblasts in the RMS and OB and an increase in the SVZ (Ribeiro Xavier et al., 2015). Additionally, genetic ablation of TAM receptor kinases Mer and Axl, which are involved with phagocytosis of apoptotic cells, result in an increase of apoptotic cells in the SVZ and RMS (Scott et al., 2001; Fourgeaud et al., 2016). Thus, it remains unclear how exactly microglia influence the SVZ, RMS, and OB niche.

Microglial roles have been investigated extensively through the use of reagents that result in their ablation. Such reagents include clodronate delivered in liposomes, which has been shown to yield a 70%–80% efficiency in microglial depletion (Torres et al., 2016; Nelson and Lenz, 2017), and Mac-1-Saporin which results in about 50% depletion of the cells (Yao et al., 2016; Han et al., 2017). Other compounds target the colony stimulating factor-1 receptor (CSF1R) expressed on microglia. CSF1R signaling is required for microglia survival, and blocking of the receptor with an antagonist results in brain wide ablation (Elmore et al., 2014). One of the compounds used is GW2580, which has been reported to inhibit CSF1R both in microglia and macrophages: when the compound was used in a model of renal and neuropsychiatric lupus lower ionized calcium binding adaptor molecule 1 (Iba1+, a marker for microglia and macrophages) counts were reported in the kidney glomeruli (Chalmers et al., 2017), but did not yield any significant change in the total number of microglial cells in normal conditions (Olmos-Alonso et al., 2016). In a model of spinal cord injury, GW2580 resulted in a 60% reduction of microglia in the lesion epicenter (Gerber et al., 2018). Another CSF1R inhibitor, BLZ945, was efficient in depleting microglia in a dose-dependent manner in the uninjured CNS, but was less effective in depleting activated microglia (Beckmann et al., 2018). A series of CSF1R inhibitors have been developed by Plexxikon Inc., which have been shown to be capable to deplete microglia. The first of these compounds was PLX3397, which in addition inhibits c-Kit (Elmore et al., 2014), and has been reported to achieve 90%–99% depletion of microglia (Szalay et al., 2016; Jin et al., 2017; Groh et al., 2019). In this study, we use a different microglial inhibitor, PLX5622, which does not act on c-Kit, and has been reported to yield ~90% microglial ablation (Acharya et al., 2016; Walter and Crews, 2017; Nissen et al., 2018; Seitz et al., 2018; Unger et al., 2018). It is delivered orally in the mouse chow, and this non-invasive delivery methods is preferred to local injections or infusion of other compounds that result in (minimal) mechanical injury in the tissue and accompanying microglial activation (Torres et al., 2016). Of clinical significance, the ablation is reversible, with almost complete repopulation of microglia in the brain by day 3 after withdrawal of PLX5622 treatment (Dagher et al., 2015). Recently PLX5622 was used to study adult neurogenesis in the OB, but not in the other SVZ-OB pathway regions. In the OB, changes in neuronal spine density and spine dynamics were seen. However, no changes were seen in neuroblast or mature neuron populations (Reshef et al., 2017). We study in this report microglia/neurogenesis interactions in all regions of SVZ-OB of adult mice. We find that microglia are not required for normal proliferation and differentiation in the adult SVZ and RMS, and we report that CSF1R inhibition *via* PLX5622 does not affect microglia proliferation rates.

## Materials and Methods

### Animals

Experiments were performed on 9-week-old male C57BL/6 mice bred in maximum isolation with 12/12 h light/dark cycle conditions. Animals had free access to food and water, and were kept on standard mice chow until experiments were started. Mice were group housed before and during experiments. All experiments were performed using protocols approved by the Stony Brook University Institutional Animal Care and Use Committee (IACUC).

### Microglia Ablation

CSF1R antagonist PLX5622 (1,200 ppm; 1,200 mg/kg) was provided by Plexxikon, and is formulated in AIN-76A chow by Research Diets. Mice were fed PLX5622 or control chow (AIN-76A) for 7 or 14 days, resulting in brain wide microglia ablation (Dagher et al., 2015).

### BrdU Administration

5-bromo-2′-deoxyuridine (BrdU, Sigma B5002) was administered in drinking water (1.0 mg/ml) supplemented with 1% sucrose to encourage drinking. Twenty five millilitters of BrdU water per mouse were given during the last 4 days of PLX5622 treatment. While the amount of water consumed was not measured, we ascertained daily that the mice had enough water. At the end of the experimental period a small volume if any of water was left. Additionally, Ki67 staining was performed on sections to further validate the BrdU results.

### Immunohistochemistry

At the 7-day (7d) or 14-day (14d) time point, mice were deeply anesthetized with 2.5% Avertin, were transcardially perfused with 25 mLs 1× phosphate buffered saline (PBS) followed by 25 mLs 4% paraformaldehyde (PFA) in 1× PBS. Brains were collected and post-fixed in 4% PFA overnight for two nights and then placed in 1× PBS until sectioned. Brains were mounted, covered in 1× PBS, and sagittal sections were collected with a Leica Vibratome VT 1000S. Tissue within 2 mm laterally of the midline were sectioned into 50 μm slices and serially collected in a 12 well plate containing 1× PBS. Plates were stored in 4°C until wells of tissue were used.

Free floating sections were incubated for 2 h in blocking solution containing 10% normal donkey (Sigma D9663) or goat serum (Southern Biotech 100241) and 0.3% Triton-x100 in 1× PBS. Immediately after blocking, tissue was incubated with primary antibodies overnight at 4°C in dilution solution (1% bovine serum albumin, 0.3% Triton-x100 in 1× PBS; Table 1). After PBS washes, sections were incubated with secondary antibodies in dilution solution for 1 h at room temp in the dark. Following another round of PBS washes, slices were mounted on slides (VWR Micro Superfrost Plus) and cover slipped (VWR Micro Cover Glass) with DAPI Fluoromount (SoutherBiotech G0100). The slides were left in the dark at room temperature overnight, and then sealed with clear nail polish. For BrdU staining, tissue was treated with 2N hydrochloric acid (HCL) for 1 h, washed with 0.1 M sodium borate, then washed with 1× PBS before treated with blocking solution.

**Table 1:** Antibodies used in experiments.

Antigen	Species	Dilution	Supplier
Iba1	Rabbit	1:500	Wako 019-19741
Sox2	Mouse	1:200	R&D Systems MAB2018
GFAP	Rabbit	1:500	Dako Z0334
Ki67	Rat	1:500	Fisher 245-564
DCX	Goat	1:200	Santa Cruz sc-8066
BrdU	Rat	1:500	Novus NB500-1690
NG2	Rabbit	1:500	Dr. Joel Levine, SBU
CC1	Mouse	1:100	CalBioChem OP80
CSF1R	Rabbit	1:200	Santa Cruz sc-692
Cleaved caspase 3	Rabbit	1:200	Cell Signaling D175
Alexa donkey anti-rat 488	Donkey	1:1,000	Life Technologies A21208
Alexa donkey anti-rabbit 647	Donkey	1:1,000	BioLegend 406414
Alexa goat anti-rabbit 488	Goat	1:1,000	Life Technologies A11008
Alexa goat anti-mouse 555	Goat	1:1,000	Life Technologies A21424
Alexa goat anti-rabbit 633	Goat	1:1,000	Life Technologies A21071
Goat anti-rat Cy5	Goat	1:1,000	Abcam ab6565

### Imaging and Analysis

A Leica SP8X confocal microscope was used for imaging. For the SVZ, tilescans (multiple consecutive images that are stitched together to make a single rectangular image) of the whole lateral wall of the SVZ on two slices per brain was imaged at 40x magnification, consistent with previous publications (Ribeiro Xavier et al., 2015). For the RMS and OB, either three 40× magnification images for the Ki67/Iba1 staining, or five 63× magnification images for the BrdU/Iba1 staining were chosen at random on slices containing the RMS and granular cell layer in the OB and marked by the confocal software. 100× magnification was used to analyze single cells. All images were obtained as 10 μm z-stacks, with 1 μm used for the z-step between each image of the z-stack. Image analysis, including cell counting, was performed on either Leica LAX-S or ImageJ (NIH) software.

### Statistical Analysis

Six mice were used per group (24 mice total), and two sections for each marker were quantified. Two-way analyses of variance (ANOVA) with Bonferroni posttest was used to evaluate data comparing the interaction between region and treatment; Student’s *t*-test was used to compare data between treatments within a single region. A *p*-value of <0.05 was used to determine significant difference with a 95% confidence interval. All graphs are plotted as averages ± SEM.

## Results

### CSF1R Inhibitor, PLX5622, Treatment Has Negligible Effect on Adult Neurogenesis

**Figure 1:**
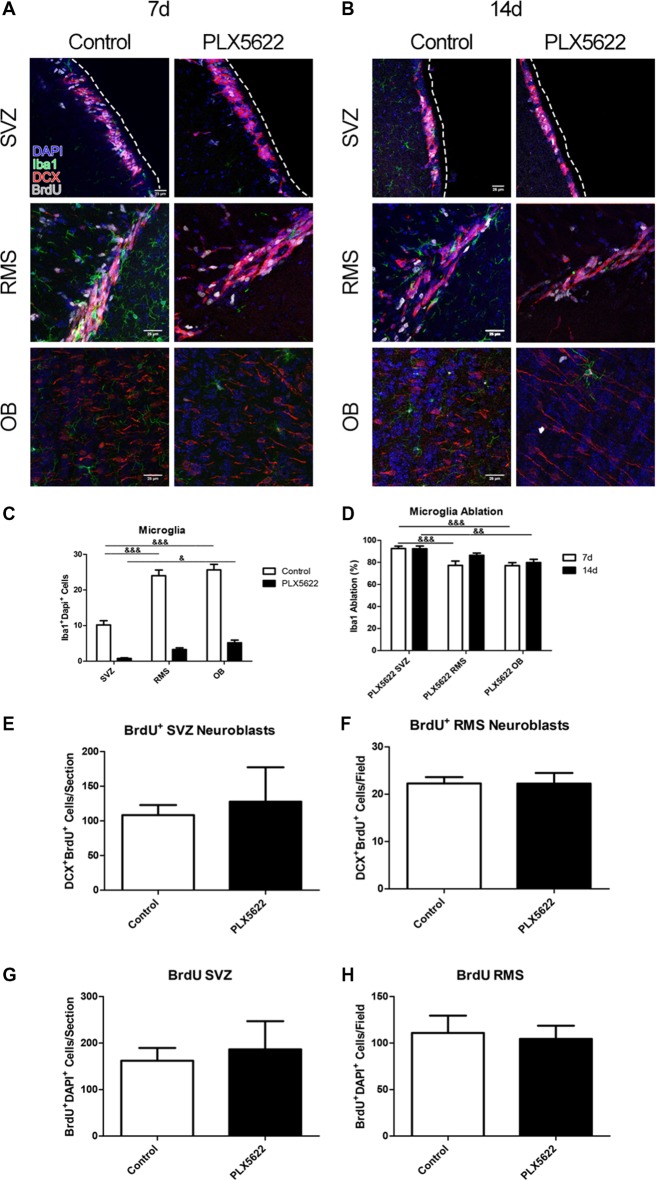
Brain wide microglia ablation results in no change on neuroblast production. Representative images of the subventricular zone (SVZ), rostral migratory stream (RMS), and olfactory bulb (OB) from 7 days (7d; **A**) and 14 days (14d; **B**) of PLX5622 treatment, with the dashed line separating the SVZ from the lateral ventricle. DAPI in blue, ionized calcium binding adaptor molecule 1 (Iba1) in green, doublecortin (DCX) in red, and 5-bromo-20-deoxyuridine (BrdU) in gray. Quantification of 14d microglia ablation shown in (**C**, *F*_(2,15)_ = 13.41, *p* = 0.0005). The ablation percentage was calculated by dividing the PLX5622 Iba1^+^ cell count for a brain from the average of the control, respective to each region (**D**, *F*_(2,15)_ = 1.56, *p* = 0.22). Quantification of 14d BrdU positive neuroblasts shown in (**E**, *p* = 0.7091) and (**F**, *p* = 0.9902), and 14d total BrdU in (**G**, *p* = 0.7168; **H**, *p* = 0.7863). ^&^ = comparison between regions within treatment. ^&^ = *p* < 0.5; ^&&^ = *p* < 0.01; ^&&&^ = *p* < 0.001. *n* = 6 for all groups, two sections per *n*. Microglia in **(C)** plotted as an average per section generated by sum of field of views.

To inquire whether the PLX5622 diet effectively ablated microglia in our system, immunofluorescence for Iba1, a marker for microglia/macrophages, was performed (Figures 1A,B, Supplementary Figure S1A). At 14 days (14d) of treatment, there were higher numbers of microglia in the OB compared to the SVZ in both control and PLX5622 treated mice, and in the RMS compared to the SVZ in control mice (Figure 1C). After 7 days (7d), microglial quantification showed significant levels of ablation compared to the respective controls in the SVZ, RMS, and OB (Supplementary Figure S1B). On average, ablation levels varied from 74% to 93%. However, the OB displayed lower levels of ablation overall compared to the SVZ after either the 7d and 14d, and in the RMS compared to the SVZ at 7d but not 14d (Figure 1D). Overall, PLX5622 treatment for 7d or 14d resulted in brain-wide microglia ablation in the SVZ, RMS, and OB. Since ablation percentages were slightly higher after 14d of PLX5622 treatment than 7d of treatment, 14d data are discussed more extensively in the subsequent experiments.

Changes in neuroblast migration were evident after microglial ablation in the SVZ *via* saporin conjugated to CD11b (Ribeiro Xavier et al., 2015). To determine if PLX5622 treatment resulted in similar changes, the newly formed and migrating neuroblasts were labeled, through co-immunostaining with doublecortin (DCX), a microtubule protein expressed in neuroblasts, and BrdU to mark neuroblasts that had been produced since its administration (Figures 1A,B). BrdU was provided to the mice in the water 4 days before the end of either the 7d or 14 time point. Mice treated with PLX5622 for 7d had less BrdU^+^ neuroblasts in the SVZ (Supplementary Figure S1C). Surprisingly, this difference no longer existed after 14d of PLX5622 treatment, and there were no differences in the RMS at either timepoint (Figures 1E,F, Supplementary Figure S1D). To account for labeling cell populations other than just neuroblasts, the numbers of all BrdU^+^ cells were counted in the SVZ and RMS (Figures 1G,H, Supplementary Figures S1E,F). Corresponding with the neuroblast counts, there was a decrease in the SVZ at 7d of PLX5622 treatment, that was no longer observed by 14d. There was no significant difference between control and PLX5622 treated mice within the RMS at either timepoint. Since the 4 days of BrdU administration may not be enough time for most BrdU labeled neuroblasts to migrate to the OB (Petreanu and Alvarez-Buylla, 2002), the analysis performed did not include the OB.

**Figure 2:**
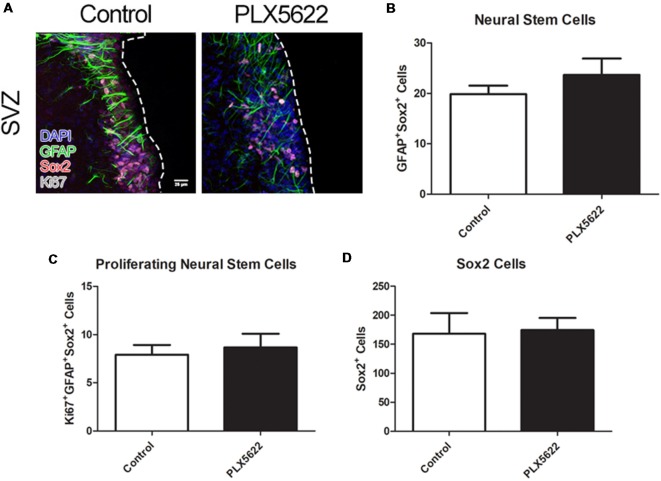
No change in neural stem cell (NSC) or TAC population from PLX5622 treatment. Representative images of the SVZ from 14d of PLX5622 treatment **(A)**, with the dashed line separating the SVZ from the lateral ventricle. DAPI in blue, glial fibrillary acidic protein (GFAP) in green, Sox2 in red, and Ki67 in gray. Quantification of NSC population shown in (**B**, *p* = 0.2465), with proliferating (Ki67+) NSCs shown in (**C**, *p* = 0.6871). Sox2+ cells, which includes NSCs and TACs, is shown in (**D**, *p* = 0.8964). *n* = 6 for all groups, two sections per *n*, plotted as per SVZ wall.

To determine if microglia ablation from PLX5622 treatment influenced the NSCs in the SVZ, staining with glial fibrillary acidic protein (GFAP) and SRY box 2 (Sox2) was performed. Additionally, Ki67, a marker for cellular proliferation, was used to label NSCs that were actively proliferating (Figure 2A, Supplementary Figure S2A). Upto 14d of PLX5622 treatment, there was no change in the numbers of NSCs in the SVZ (Figure 2B, Supplementary Figure S2B); nor was there any change in the numbers of proliferating NSCs following PLX5622 treatment (Figure 2C, Supplementary Figure S2C). As TACs express Sox2, total SVZ Sox2 was quantified; no changes in the numbers of Sox2^+^ cells in the SVZ after PLX5622 treatment were evident (Figure 2D, Supplementary Figure S2D).

**Figure 3:**
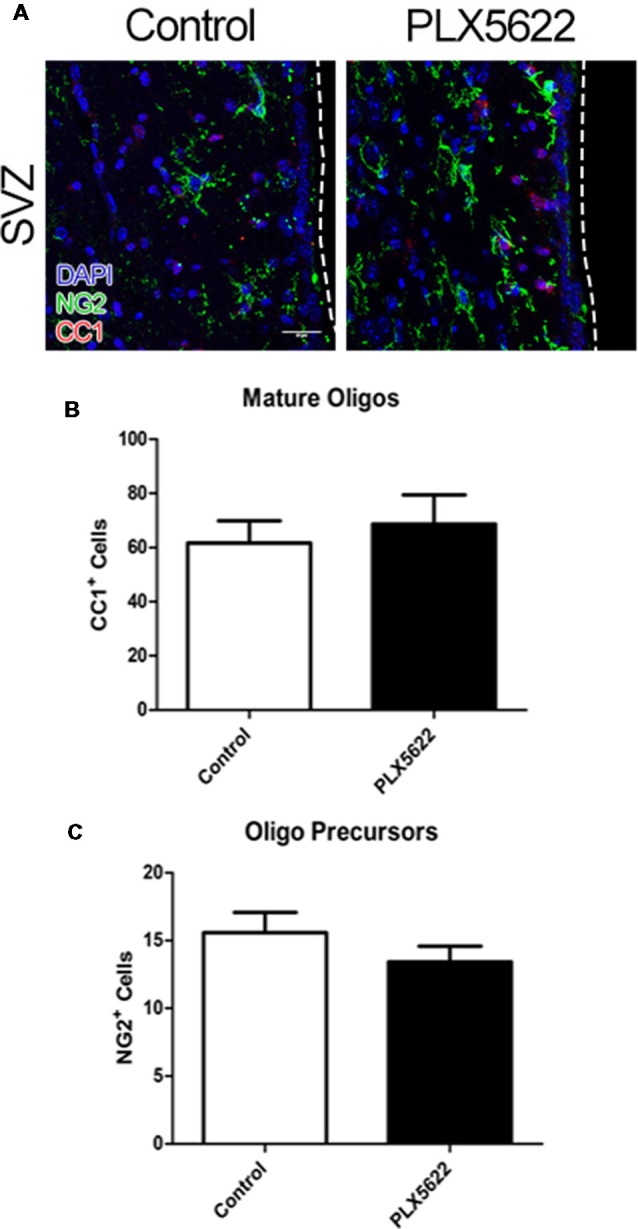
No change in oligo precursors and mature oligos from PLX5622 treatment. Representative images of the SVZ from 14d of PLX5622 treatment **(A)**, with the dashed line separating the SVZ from the lateral ventricle. DAPI in blue, neural/glial antigen 2 (NG2) in green, and CC1 in red. Quantification of mature oligos in (**B**, *p* = 0.6101) and oligo precursors in (**C**, *p* = 0.2670). *n* = 6 for all groups, two sections per *n* plotted as per SVZ wall.

NSCs in the adult SVZ may also differentiate into oligodendrocytes (oligos), but in lower numbers than TACs/neuroblasts. This differentiation is typically observed on the superior wall of the SVZ, but not in the lateral wall (Menn et al., 2006; Ortega et al., 2013). To examine if microglia influence adult oligogenesis in the SVZ, a marker for mature oligos [anti-adenomatous polyposis coli clone CC1 (CC1)] and neural/glial antigen 2 (NG2), a marker for oligo progenitors, were used to image the superior wall of the SVZ (Figure 3A, Supplementary Figure S3A). Again, there were no differences after 14 or 7d of PLX5622 treatment compared to control in either numbers of mature oligos or oligo precursor cells (Figures 3B,C, Supplementary Figures S3B,C).

As mentioned earlier, TAM receptor ablation has resulted in increased numbers of apoptotic cells. We therefore stained for cleaved caspase 3 (CC3) to investigate levels of apoptosis in the SVZ, RMS, and the dentate gyrus (DG) of the hippocampus, where apoptotic cells are reported to be cleared by microglia (Sierra et al., 2010). Surprisingly, there was no increase in apoptotic cells in any of these regions after PLX5622 treatment (Supplementary Figure S4). Taken together, these data suggest that microglia may not be required for normal adult neurogenesis in the SVZ niche.

### CSF1R Inhibition *via* PLX5622 Has No Effect on Microglia Proliferation Rates

**Figure 4:**
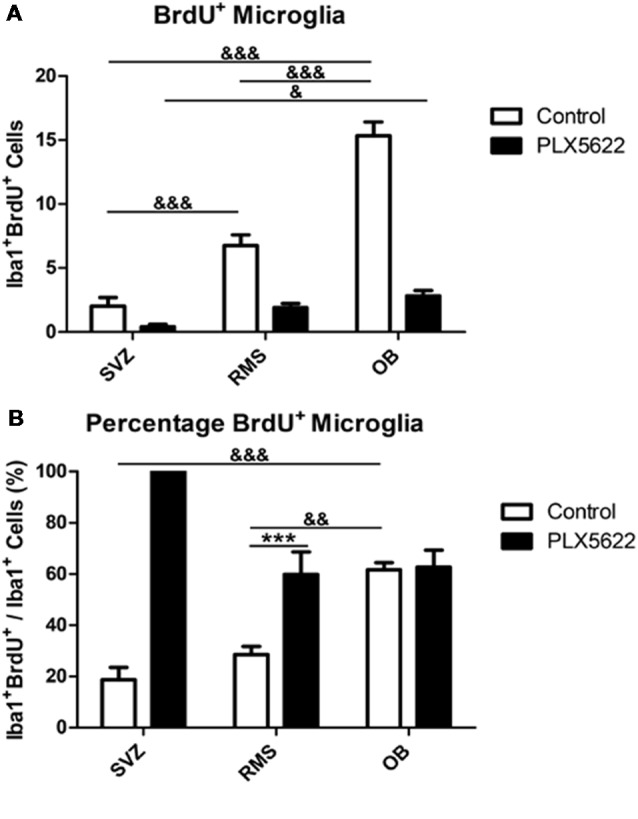
Remaining microglia have no change in rate of replication after PLX5622 treatment. BrdU^+^Iba1^+^ cells were quantified (**A**, *F*_(2,15)_ = 30.39, *p* < 0.0001) and normalized in (**B**, *F*_(2,15)_ = 51.75, *p* < 0.0001). The OB contained more BrdU^+^ microglia than the RMS and SVZ in control mice, but only more than the SVZ in PLX5622 treated mice. When normalized, control OBs had a higher percentage of BrdU^+^ microglia compared to the RMS and SVZ, with no significant difference between control and PLX5622 treated mice. ^&^ = comparison between regions within treatment, * = comparison between treatments within a region. */^&^ = *p* < 0.5; **/^&&^ = *p* < 0.01; ***/^&&&^ = *p* < 0.001. *n* = 6 for all groups, two sections per *n*. Microglia in **(A)** plotted as an average per section generated by sum of field of views.

To understand better whether CSF1R inhibition by PLX5622 affected resting or active/proliferating microglia in the SVZ niche, cells were co-labeled with BrdU and Iba1 (Figures 1A,B). The total number of microglia counted for control SVZ, RMS, and OB were 122, 288, and 308, respectively. For PLX5622 SVZ, RMS, and OB, 9, 39, and 62 cells were counted. BrdU^+^ microglia were quantified as having punctate BrdU labeling in the microglia somata, as reported by others (Tay et al., 2017). Interestingly, the control OB had more microglia labeled with BrdU than the SVZ and RMS (Figure 4A, Supplementary Figure S5A; the number of microglia that were BrdU^+^ in control SVZ, RMS, and OB were 24, 81, and 184, whereas for PLX5622 SVZ, RMS, and OB, there were 9, 23, and 34 double positive cells). Despite being so few microglia remaining after PLX5622 treatment, all treated regions had microglia that expressed BrdU, including the OB which contained significantly more residual microglia than the SVZ. To normalize the different regions, the percentage of BrdU^+^ microglia was generated by dividing BrdU^+^ microglia by the total number of microglia in the region for each brain; the numbers were then averaged (Figure 4B, Supplementary Figure S5B). Surprisingly, PLX5622 treated mice had no significant change in the percentage of BrdU^+^ microglia from control mice in the OB, but there was a significant increase in the RMS compared to control. Additionally, the OB had an almost identical percentage of BrdU^+^ microglia in both control and PLX5622 treated mice. The PLX5622 SVZ contained more BrdU^+^ microglia than control animals, but the difference was not statistically significant (since the very low numbers of residual microglia in the SVZ after PLX5622 treatment was not evenly distribute amongst the six animals counted, which rendered the statistics not reliable).

**Figure 5:**
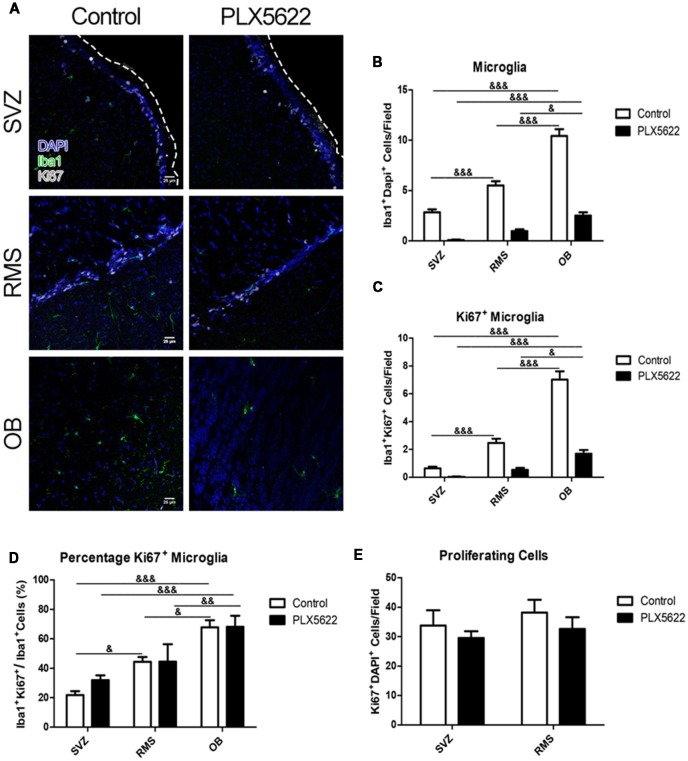
Treatment with PLX5622 has no effect on microglia proliferation. Representative images of the SVZ, RMS, and OB from 14d of PLX5622 treatment **(A)**, with the dashed line separating the SVZ from the lateral ventricle. DAPI in blue, Iba1 in green, Ki67 in gray. Quantification of microglia ablation shown in (**B**, *F*_(2,15)_ = 55.46, *p* < 0.0001). Ki67^+^Iba1^+^ cells were counted (**C**, *F*_(2,15)_ = 64.52, *p* < 0.0001) and normalized (**D**, *F*_(2,15)_ = 12.77, *p* = 0.0006). Similar to the BrdU data, the OB contained more Ki67^+^ microglia than the RMS and SVZ in both control and PLX5622 treated mice. Total Ki67^+^ cells from the SVZ and RMS were quantified in (**E**, *F*_(2,15)_ = 0.11, *p* = 0.74). & = comparison between regions within treatment, ^&^ = *p* < 0.5; ^&&^ = *p* < 0.01; ^&&&^ = *p* < 0.001. *n* = 6 for all groups.

To validate this result, we also quantified Ki67-labeled microglia in the SVZ, RMS, and OB (Figure 5A, Supplementary Figure S6A). Total microglia counted for control SVZ, RMS, and OB were 109, 199, and 375, respectively. For PLX5622 SVZ, RMS, and OB, 3, 35, and 90 cells were counted. Iba1+ Ki67+ microglia were again counted for each region (Figure 5B, Supplementary Figure S6B). As with the BrdU, microglia co-labeled with punctate Ki67 were detected (the number of microglia that were Ki67^+^ in control SVZ, RMS, and OB were 23, 89, and 253, whereas for PLX5622 SVZ, RMS, and OB, there were 1, 19, and 61 double positive cells). Upon quantification, similar results were found, with the OB having the most Ki67^+^ microglia, followed by the RMS (Figure 5C, Supplementary Figure S6C). As with BrdU labeling, PLX5622 treated OB had an almost identical percentage of Ki67^+^ microglia as control (Figure 5D, Supplementary Figure S6D). There were no significant differences between control and PLX5622 treated mice at 14d, but there was an increase in PLX5622 treated mice at 7d in the RMS. The discrepancies in numbers of Iba1+ BrdU+ and Iba1+ Ki67+ in the RMS data could be attributed to very low overall number of remaining microglia (Iba1+) positive cells from after PLX5622 treatment. Total Ki67^+^ cells were counted in both the SVZ and RMS (Figure 5E, Supplementary Figure S6E). As with BrdU, very few Ki67^+^ cells were found in the OB and almost all that were found were microglia (Figure 5A, Supplementary Figure S6A). There were fewer proliferating cells after PLX5622 treatment, but similar to the total BrdU^+^ cells, these changes were not significant.

To confirm that the microglia in the OB are sensitive to and would be targeted by the CSF1R inhibitor, CSF1R staining was performed (Supplementary Figures S7A,B). Both the control and PLX5622-treated brains presented with CSF1R expressing cells. 100× magnification images were taken of CSF1R^+^ cells, which had the same shape and structure as ramified microglia. Ki67 labeling was done, and the punctate staining pattern that was seen from BrdU and Ki67 staining with Iba1 was observed as before (Sobecki et al., 2016; Tay et al., 2017). The 100× magnification also helped to ascertain that the Ki67 signal was localized in the cell soma. Taken together, these data suggest that although CSF1R stimulation/signaling is needed for microglia survival, it is not needed for microglia proliferation and replication.

## Discussion

While microglia may influence SVZ adult neurogenesis, the results of the present study suggest that microglia are not required for normal proliferation and differentiation of this process *in vivo*. We discovered that NSCs populations are not affected by up to 14d of PLX5622 treatment (Figures 2B,C). Additionally by 14d of treatment, there was no change in neuroblast production (Figure 1E), nor were there changes in oligogenesis (Figure 3). To our knowledge, this is the first study to look at SVZ niche adult neurogenesis after *in vivo* brain-wide microglia ablation.

In the adult hippocampus, microglia are reported as required to assist in normal, physiological neurogenesis (Sierra et al., 2010). Using a different method of ablation, other labs have locally ablated microglia around the SVZ niche and reported aberrant neuroblast production/migration (Ribeiro Xavier et al., 2015). While we did not explore the hippocampus, our results do not suggest that microglia are needed for normal neuroblast production, migration out of the SVZ, or migration through the RMS. One possible explanation for this is the presence of the supportive “tube” of astrocytes that surround the RMS (Lois et al., 1996) that may be able to substitute for the loss of microglia. Treatment with PLX3397, another compound containing a CSF1R inhibitor, for 7 days resulted in an increase of GFAP mRNA levels, although no difference in astrocyte cell counts were evident (Elmore et al., 2014). Another possible explanation is that, unlike in the hippocampus, phagocytosis of neuroblasts is not a required step for normal SVZ-OB adult neurogenesis. This is supported by the finding that there was no change in the total number of BrdU^+^ cells or Ki67^+^ cells in SVZ and RMS up to 14d of PLX5622 treatment (Figures 1F, 5E). Lastly, the reported change in neuroblast production/migration from saporin—CD11b focal microglia ablation could be due to the release of pro-inflammatory cytokines around the SVZ (Ribeiro Xavier et al., 2015). PLX3397 and PLX5622 treatments have been shown to not result in changes in microglia related cytokine release, such as TNF-α and IL-1β (Elmore et al., 2014; Reshef et al., 2017), thus accounting for a possible difference between these two experimental setups.

One of the main functions of microglia at homeostasis is the phagocytosis and management of neuronal synapses in the OB (Wake et al., 2013). A recent study using a genetic model to specifically ablate Mer and Axl on microglia, which are important receptors for microglial phagocytosis, reported an increased number of BrdU+ cells in the OB and a higher cell density in the granule and glomerular cell layers. Many of these cells were NeuN+ positive, but others were not (Fourgeaud et al., 2016). In contrast, using PLX5622 to ablate microglia in the OB, no increase in BrdU positive cells in the OB was seen. Instead, changes in dendritic spines dynamics and lower spine density were observed (Reshef et al., 2017). Another group reported no changes in caspase-3 gene expression using RT-PCR data after 3 weeks of PLX5622 treatment (Walter and Crews, 2017). Similarly, we found no change in the number of apoptotic cells in the SVZ or RMS after 14d of treatment (Supplementary Figure S4). The differences among these reports remain unclear. One possible explanation may be that there are increased numbers of other cell types (GFAP+ or oligo precursors) that are expressing BrdU in the OB; if so, further characterization of the OB after the various microglial ablation methods is warranted. Regardless, these reports suggest that at brain homeostasis, microglia in the OB may have an important active role in regulating neural circuits. In support of this, microglia in the OB have a higher turnover rate than other regions of the brain, including the cortex and the hippocampus (Tay et al., 2017). Interestingly, our data show that PLX5622 treatment was not as effective in the OB compared to the SVZ (Figure 1D). Accordingly, the PLX5622 treated OB had no differences in BrdU^+^ or Ki67^+^ microglia compared to control (Figures 4B, 5D). It is possible that, due to their high activity at homeostasis in the OB, microglia are stimulated *via* a route different than CSF1R stimulation. However, another likely explanation is that the turnover rate of OB microglia is high enough to partially compensate for the PLX5622 CSF1R antagonist. With PLX5622, our data suggest that CSF1R inhibition does not affect the rate of remaining microglia proliferation/turnover (Figures 4B, 5D). The remaining microglia still express CSF1R (Supplementary Figures S6A,B), and would therefore be susceptible to PLX5622. A recent discovery is that microglia repopulation after PLX5622 treatment ensues from the remaining resident populations of microglia after ablation, and not from a progenitor or peripheral source (Huang et al., 2018), also suggesting that the remaining microglia are still capable to proliferate. A different CSF1R inhibitor, the tyrosine kinase inhibitor GW2580, has been reported to decrease CSF1R+ cell proliferation. However, GW2580 does not cause microglia ablation (Olmos-Alonso et al., 2016).

In conclusion, the results presented here show that PLX5622-induced brain-wide ablation of microglia for up to 14 days does not significantly modify SVZ-OB adult neurogenesis. CSF1R inhibition *via* PLX5622 did not change microglia proliferation rates in the SVZ, RMS, or OB. The microglial population in the OB is unique compared to the SVZ and RMS in its ability to compensate for the CSF1R inhibition. These findings are important for furthering our understanding of microglia in specific regions of the brain, and how responsive local neurogenesis/microglia may be if targeted in cases of brain disease or injury.

## Data Availability

All datasets generated for this study are included in the manuscript and/or the Supplementary Files.

## Author Contributions

JK designed and carried out the experiments, collected and analyzed the data, and wrote drafts and edited the manuscript. MW and SG collected and analyzed the data. ST designed the experiments, edited drafts of the manuscript.

## Conflict of Interest Statement

The authors declare that the research was conducted in the absence of any commercial or financial relationships that could be construed as a potential conflict of interest.
