# Supplementary material for: Proliferation and Differentiation in the Adult Subventricular Zone Are Not Affected by CSF1R Inhibition
Source: Front Cell Neurosci. 2019 Apr 2;13:97. doi: 10.3389/fncel.2019.00097 (PMC6454047; doi:10.3389/fncel.2019.00097)
Supplement: Supplementary file 1 [file Data_Sheet_1.pdf]

*Supplementary Figure 1*

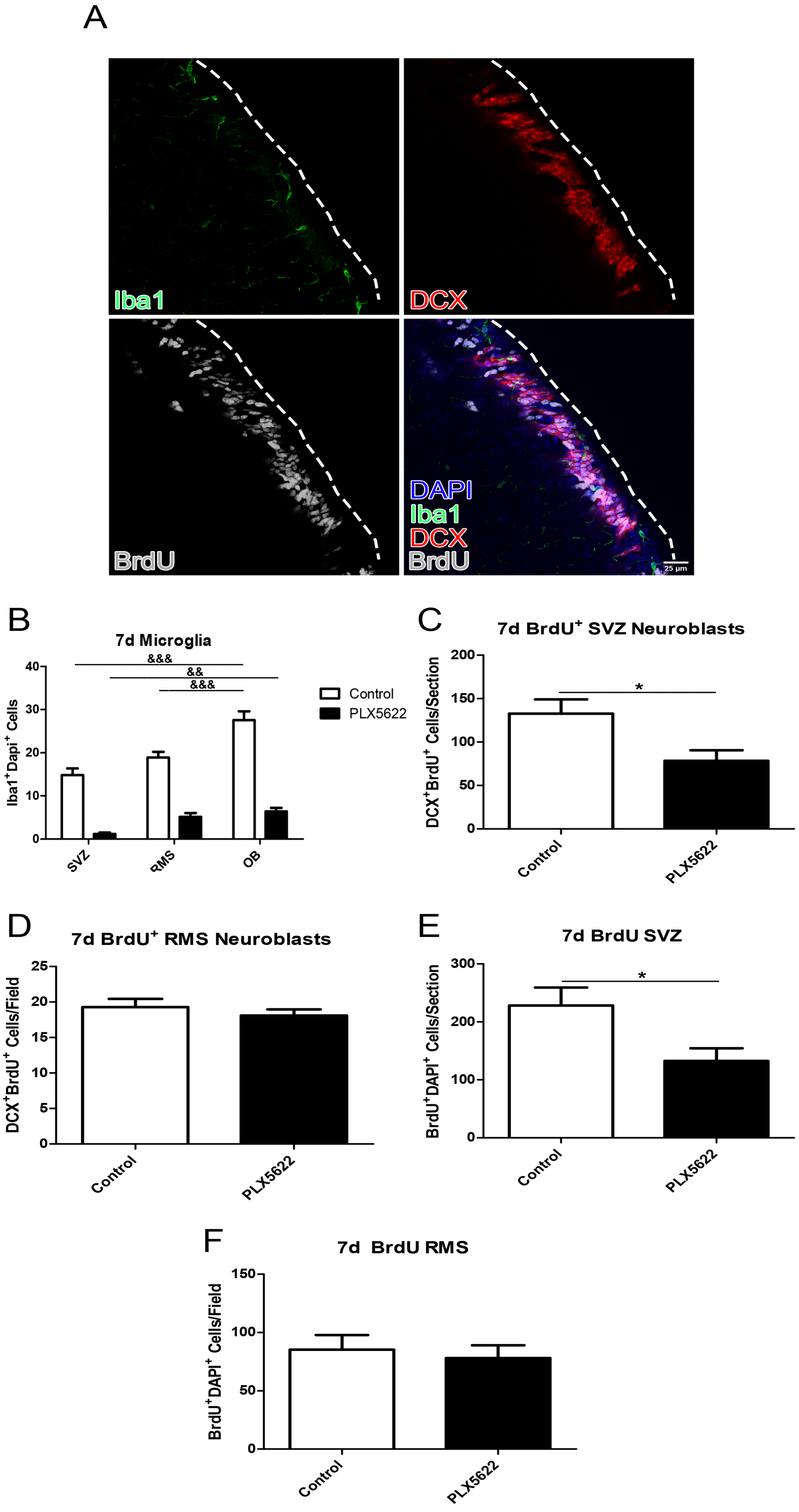

**Supplementary Figure 1: Brain Wide Microglia Ablation Results in No Change on Neuroblast Production**. Representative single channel and composite image shown of the 7d SVZ (A) with the dashed line separating the SVZ from the lateral ventricle. DAPI in blue, Iba1 in green, DCX in red, and BrdU in grey. Quantification of 7d microglia ablation shown in (B, $F(2,15) = 7.99$, $p = 0.0045$). Quantification of 7d BrdU positive neuroblasts in the SVZ (C, $p = 0.039$) and RMS (D, $p = 0.4471$), and total BrdU in the SVZ (E, $p = 0.024$) and RMS (F, $p = 0.6691$). & =comparison between regions within treatment, * =comparison between treatments within a region. */& = $p$ <0.5; **/&& = $p$ <0.01; ***/&&& = $p$ <0.001. n=6 for all groups, 2 sections per n. Microglia in (B) plotted as an average per section generated by sum of field of views. Scale bar: 25um.

*Supplementary Figure 2*

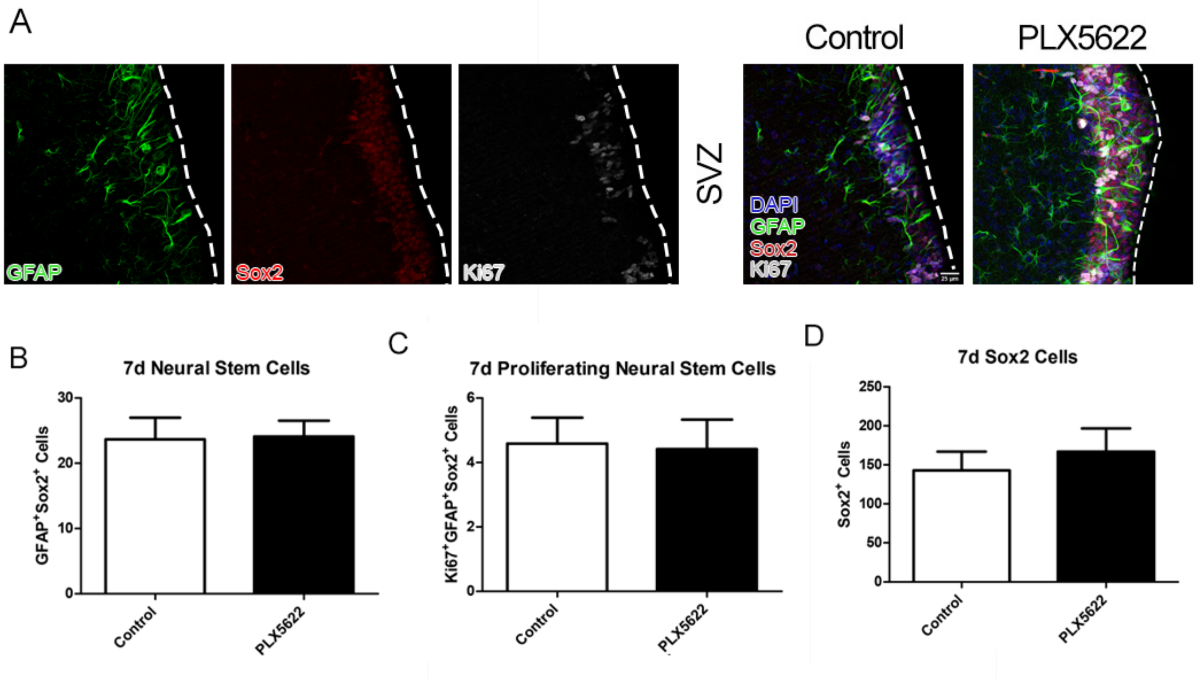


**Supplementary Figure 2: No Change in NSC or TAC Population from 7 days of PLX5622 Treatment.** Representative single channel and composite images shown of the 7d SVZ (A), with the dashed line separating the SVZ from the lateral ventricle. DAPI in blue, GFAP in green, Sox2 in red, and Ki67 in grey. Quantification of NSC population shown in (B, p=0.9204), with proliferating (Ki67+) NSCs shown in (C, p=0.8929). Sox2+ cells, which includes NSCs and TACs, is shown in (D, p=0.5353). n=6 for all groups, 2 sections per n., plotted as per SVZ wall. Scale bar: 25um.

*Supplementary Figure 3*

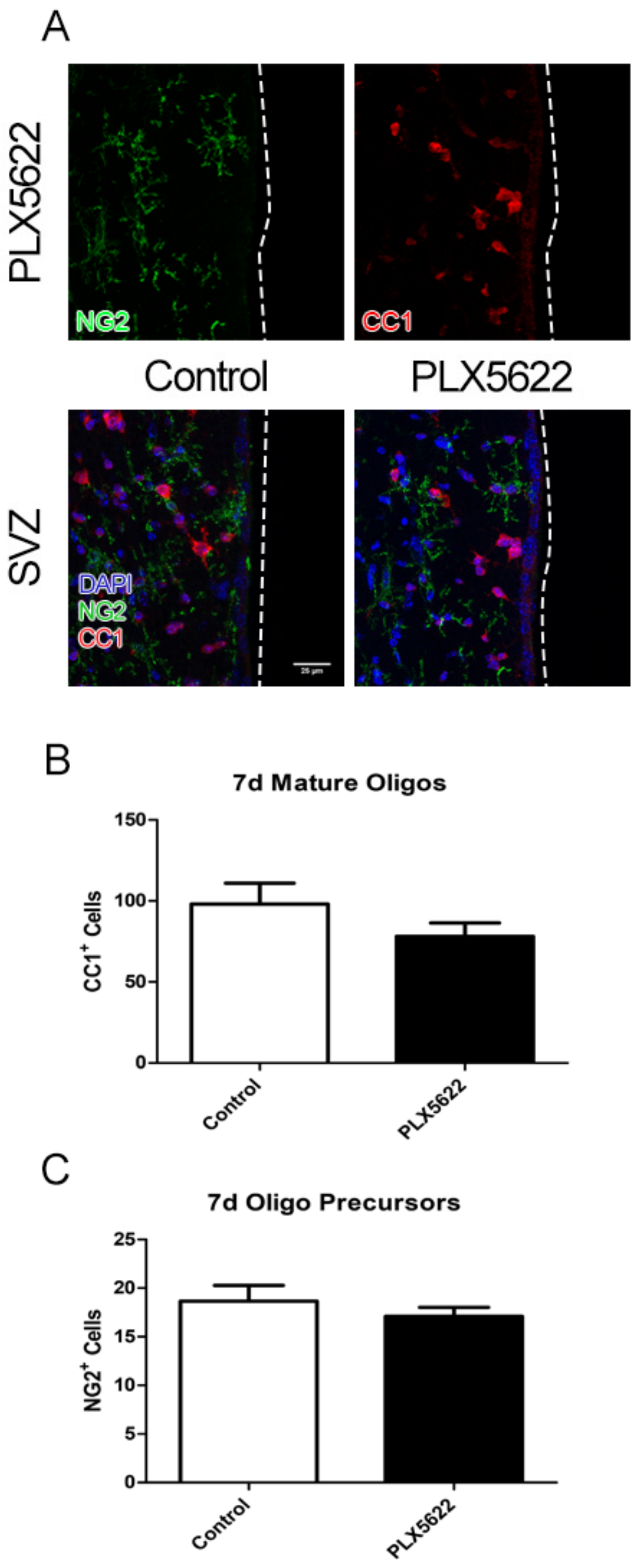

**Supplementary Figure 3: No Change in Oligo Precursors and Mature Oligos from 7 days of PLX5622 Treatment.** Representative single channel and composite images shown of the 7d SVZ (A), with the dashed line separating the SVZ from the lateral ventricle. DAPI in blue, NG2 in green, and CC1 in red. Quantification of mature oligos in (B, p=0.2037) and oligo precursors in (C, p=0.4073). n=6 for all groups., 2 sections per n., plotted as per SVZ wall. Scale bar: 25um.

*Supplementary Figure 4*

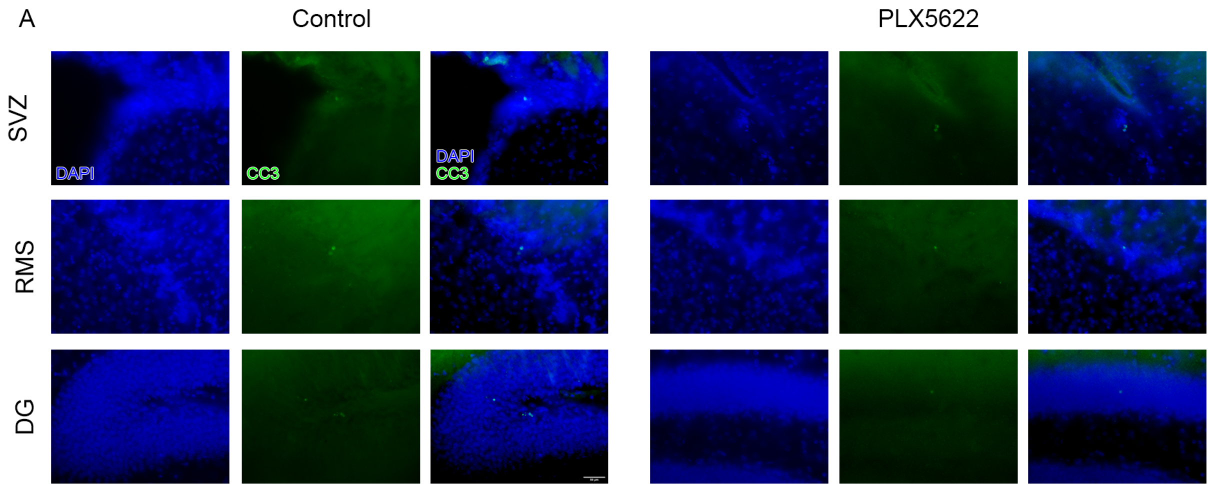


**Supplementary Figure 4: No Change in Apoptotic Cells after 14d of PLX5622 Treatment.** Representative single channel and composite images of the SVZ, RMS, and dentate gyrus (DG) (A). DAPI in blue, cleaved caspase 3 in green. No increase was found in reported areas of microglial clearing of apoptotic cells after PLX5622 treatment.

*Supplementary Figure 5*

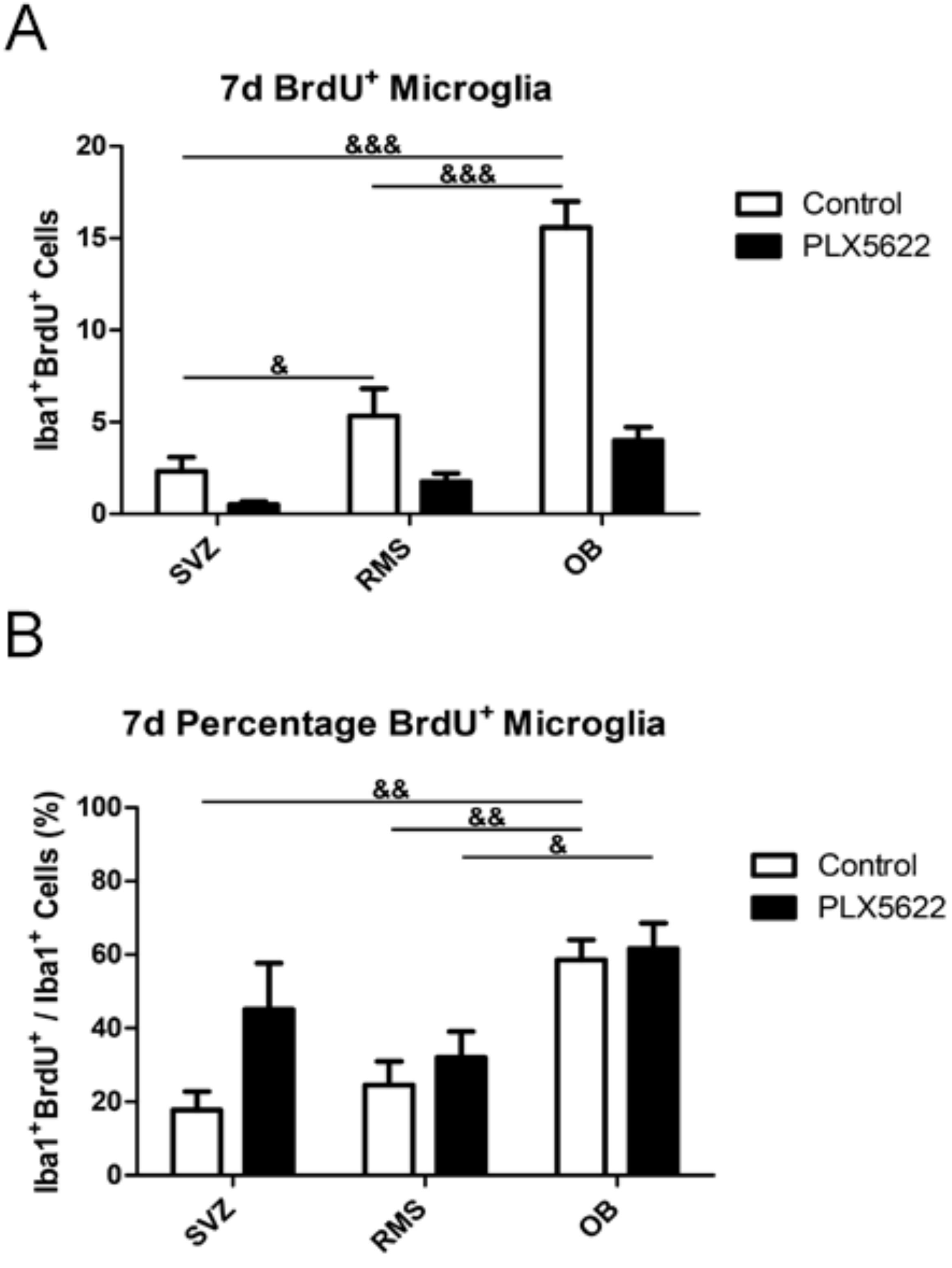

**Supplementary Figure 5: Remaining Microglia have No Change in Rate of Replication After 7 days of PLX5622 Treatment.** $BrdU^{+}Iba1^{+}$ cells were quantified (A, $F(2,15) = 13.80$, $p = 0.0004$) and normalized in (B, $F(2,15) = 1.85$, $p = 0.19$). The OB contained more $BrdU^{+}$ microglia than the RMS and SVZ in control mice. When normalized, control OBs had a higher percentage of $BrdU^{+}$ microglia compared to the RMS and SVZ, with no significant difference between control and PLX5622 treated mice. & =comparison between regions within treatment, * =comparison between treatments within a region. */& = $p$ <0.5; **/&& = $p$ <0.01; ***/&&& = $p$ <0.001. n=6 for all groups, 2 sections per n. Microglia in (A) plotted as an average per section generated by sum of field of views.

*Supplementary Figure 6*

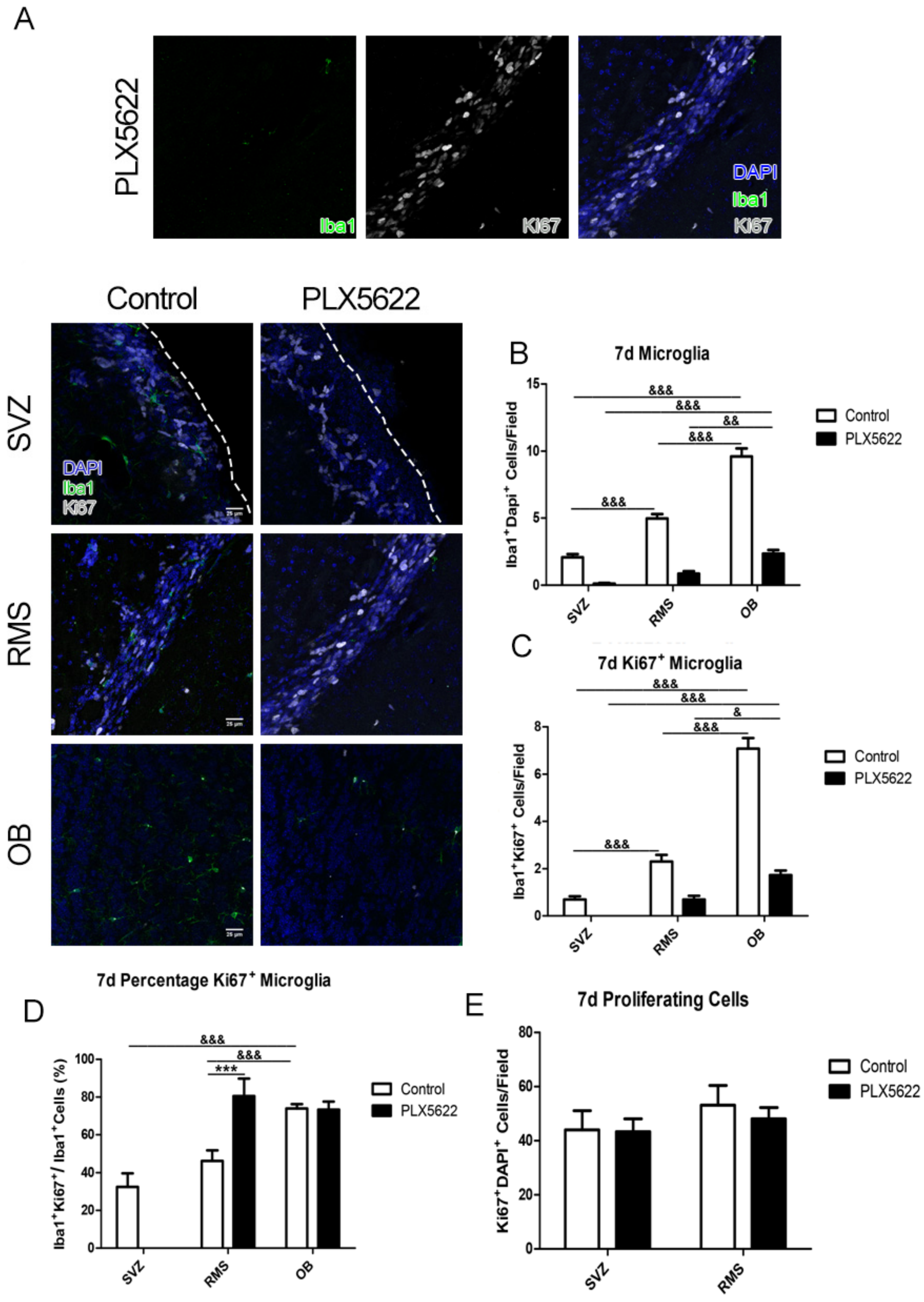

**Supplementary Figure 6: Treatment with PLX5622 for 7 days has No Effect on Microglia Replication.** Representative single channel and composite images of the SVZ, RMS, and OB (A), with the dashed line separating the SVZ from the lateral ventricle. DAPI in blue, Iba1 in green, Ki67 in grey. Quantification of microglia ablation shown in (B, $F(2,15) = 55.46$, $p < 0.0001$). $Ki67^{+}Iba1^{+}$ cells were counted (C, $F(2,15) = 64.52$, $p < 0.0001$) and normalized (D, $F(2,15) = 12.77$, $p = 0.0006$). Similar to the BrdU data, the OB contained more $Ki67^{+}$ microglia than the RMS and SVZ in both control and PLX5622 treated mice. Total $Ki67^{+}$ cells from the SVZ and RMS were quantified in (E, $F(2,15) = 0.11$, $p = 0.74$). & =comparison between regions within treatment, * =comparison between treatments within a region. */& = $p$ <0.5; **/&& = $p$ <0.01; ***/&&& = $p$ <0.001. n=6 for all groups, 2 sections per n.

*Supplementary Figure 7*

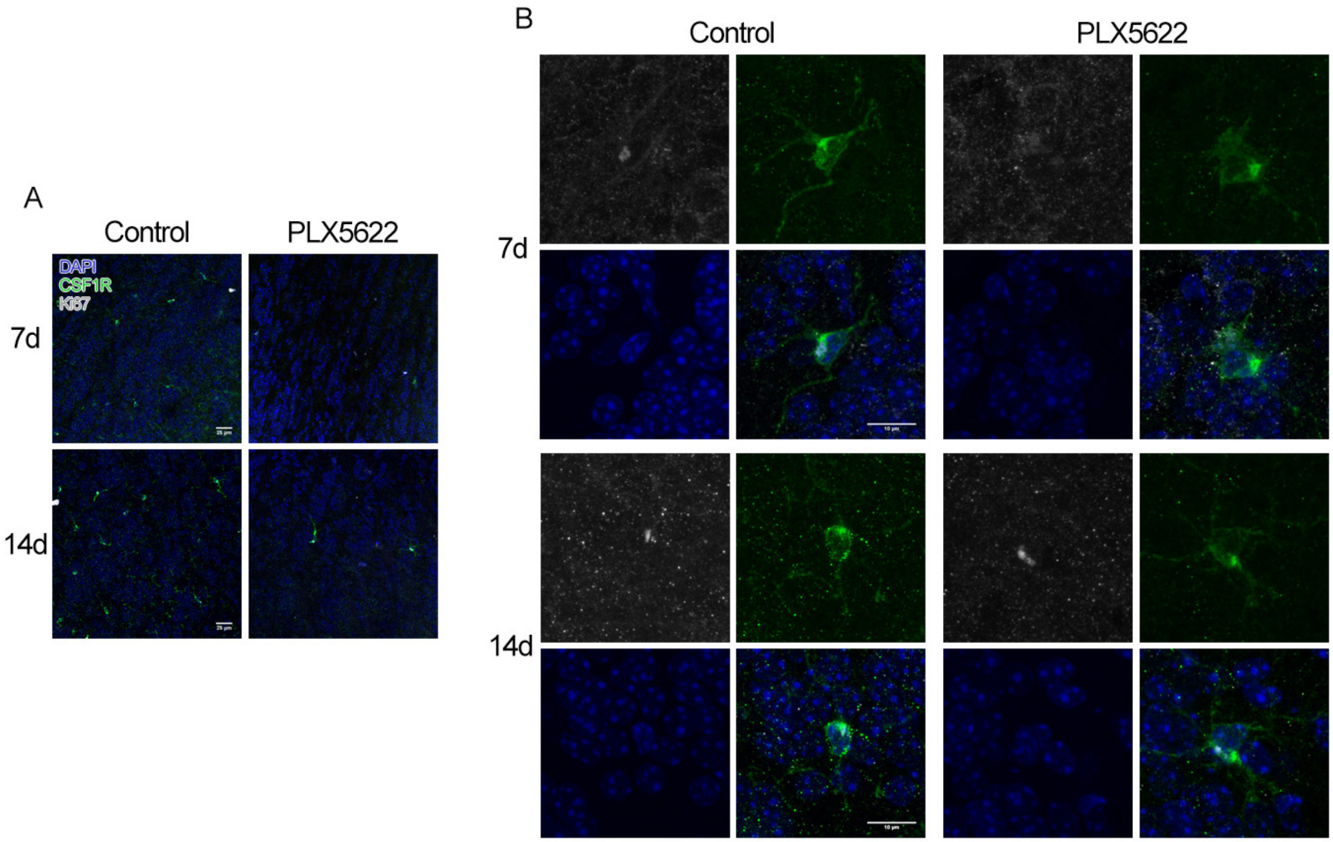


**Supplementary Figure 7: Microglia Remaining in the Olfactory Bulb after PLX5622 Treatment Express CSF1R and Ki67.** Representative images of the OB from 7d and 14d (A). DAPI in blue, Iba1 in green, and Ki67 in grey. High magnification images shown in (B). Ki67 was found in CSF1R$^{+}$ cells in the OB up to 14d of PLX5622 treatment.
